# Dietary Ellagic Acid Protects and Ameliorates Copper Sulfate-Induced Toxicity in Common Carp (*Cyprinus carpio*)

**DOI:** 10.3390/vetsci13080809

**Published:** 2026-08-15

**Authors:** Serpil Mişe Yonar, Mehmet Nuri Çakmak, Duygu Tanrıkulu, Mücahit Eroğlu, Muhammet Enis Yonar

**Affiliations:** 1 Department of Aquaculture, Faculty of Fisheries, Firat University, Elazig 23200, Türkiye; s_mise@firat.edu.tr (S.M.Y.); mncakmak@firat.edu.tr (M.N.Ç.); 2 Department of Biology, Faculty of Arts and Sciences, Kafkas University, Kars 36100, Türkiye; duygutanrikulu@kafkas.edu.tr; 3 Department of Basic Aquatic Sciences, Faculty of Fisheries, Firat University, Elazig 23200, Türkiye; meroglu44@firat.edu.tr

**Keywords:** antioxidant defense, copper sulfate, fish, ellagic acid, hematology, immunity, oxidative stress

## Abstract

Copper sulfate (CuSO_4_) is one of the most widely used chemotherapeutic agents in aquaculture and aquatic veterinary medicine because of its broad-spectrum antiparasitic, antifungal, and antimicrobial activities. However, repeated exposure may induce oxidative stress and impair hematological and immune functions, thereby compromising fish health. This study evaluated the effects of pre-exposure and post-exposure dietary ellagic acid (EA) supplementation on CuSO_4_-induced hematological, immunological, and oxidative stress alterations in common carp (*Cyprinus carpio*). A total of 150 fish were randomly allocated to five experimental groups: Control, EA, CuSO_4_, pre-exposure EA + CuSO_4_, and post-exposure CuSO_4_ + EA. Fish were fed a diet supplemented with 100 mg EA kg^−1^ feed for 14 days either before or after repeated CuSO_4_ exposure (1 mg L^−1^, 30 min day^−1^ for seven consecutive days). Hematological variables, innate immune responses, and oxidative stress biomarkers were subsequently evaluated. CuSO_4_ exposure significantly decreased red blood cell (RBC) count, hemoglobin (Hb), hematocrit (Ht), phagocytic activity (PA), phagocytic index (PI), total plasma protein (TP), immunoglobulin M (IgM), bactericidal activity (BA), lysozyme (LYZ), superoxide dismutase (SOD), catalase (CAT), glutathione peroxidase (GPx), glutathione S-transferase (GST), and reduced glutathione (GSH), while significantly increasing mean corpuscular hemoglobin concentration (MCHC), white blood cell (WBC) count, respiratory burst activity (NBT), myeloperoxidase (MPO), and malondialdehyde (MDA) (*p* < 0.05). Dietary EA supplementation markedly alleviated these alterations by restoring hematological homeostasis, improving innate immune responses, reducing lipid peroxidation, and reinforcing antioxidant defense systems in the liver, kidney, spleen, and gill. Both pre-exposure and post-exposure EA supplementation were associated with improvements in the evaluated biomarkers under the experimental conditions of the present study. However, because the two supplementation strategies were not evaluated against a time-matched CuSO_4_ recovery control group, the present study does not permit a direct comparison of their relative efficacy. These findings demonstrate that dietary EA was associated with improvements in hematological, immunological, and oxidative stress biomarkers following experimental CuSO_4_ exposure, potentially through antioxidant and immunomodulatory mechanisms. Therefore, dietary EA may serve as a promising functional feed additive to support antioxidant and immune status during experimental CuSO_4_ exposure in common carp. Further studies including pathogen challenge models are required before its use as an adjunct to CuSO_4_ treatments can be recommended.

## Simple Summary

Copper sulfate is widely used in aquaculture to control parasites, fungi, and bacterial infections. However, repeated exposure may also damage fish health by weakening the immune system and increasing oxidative stress. Natural feed additives with antioxidant properties may help reduce these adverse effects. In this study, we investigated whether dietary ellagic acid, a naturally occurring plant polyphenol, could protect common carp against copper sulfate-induced toxicity. Fish receiving ellagic acid showed improved blood health, stronger innate immune responses, enhanced antioxidant defenses, and reduced oxidative damage compared with fish exposed to copper sulfate alone. Moreover, administering ellagic acid before copper sulfate exposure provided greater protection than supplementation after exposure. These findings suggest that dietary ellagic acid may be a practical nutritional strategy to improve fish health and reduce the adverse effects of copper sulfate treatment in aquaculture.

## 1. Introduction

The rapid expansion of aquaculture has substantially increased demand for effective disease prevention and health management strategies to ensure sustainable fish production [1]. Intensive farming practices, characterized by high stocking densities and frequent environmental fluctuations, increase cultured fish susceptibility to parasitic, fungal, bacterial, and opportunistic diseases, resulting in considerable economic losses worldwide [2]. Consequently, disease prevention and post-exposure intervention have become fundamental components of modern aquaculture and aquatic veterinary practice, with chemotherapeutic agents playing an essential role in controlling infectious diseases, safeguarding animal welfare, reducing mortality, and maintaining production efficiency [3]. Nevertheless, the success of chemotherapeutic treatments depends not only on their therapeutic efficacy but also on their safety, since repeated or inappropriate applications may compromise fish physiology, impair welfare, and ultimately reduce treatment success and production sustainability [2,3].

Among chemotherapeutic agents used in aquaculture and veterinary medicine, copper sulfate (CuSO_4_) remains one of the most widely used compounds because of its broad- spectrum antiparasitic, antifungal, antimicrobial, and algicidal activities. Owing to its low cost, wide availability, ease of application, and proven therapeutic efficacy, CuSO_4_ is routinely used in freshwater aquaculture to treat and prevent ectoparasitic infestations, saprolegniasis, bacterial gill diseases, and excessive algal proliferation, making it an indispensable tool in aquatic veterinary therapeutics and fish health management [4,5]. In addition to its therapeutic applications, CuSO_4_ is widely employed as a disinfectant and water-quality management agent to reduce pathogen loads and improve environmental conditions in culture systems [5]. Despite these considerable advantages, CuSO_4_ has a relatively narrow therapeutic safety margin, and its toxicity is strongly influenced by environmental factors, including water hardness, alkalinity, pH, temperature, and organic matter content, which markedly affect copper bioavailability [5,6,7]. Experimental evidence has demonstrated that excessive or repeated exposure to CuSO_4_ induces hematological disturbances, oxidative stress, inflammatory responses, serum biochemical alterations, histopathological lesions, and damage to vital organs, including the intestine and liver [8,9,10,11]. Furthermore, environmentally relevant concentrations of CuSO_4_ have been shown to disrupt molecular signaling pathways and cellular homeostasis, suggesting that even sublethal exposure may adversely affect fish health under aquaculture conditions [12]. Therefore, increasing attention has been directed toward identifying complementary nutritional strategies that reduce CuSO_4_-induced physiological disturbances without compromising its therapeutic efficacy. In this context, several natural bioactive compounds, including vitamin E, eucalyptol, and Spirulina, have demonstrated promising protective effects against copper- induced toxicity in fish [13,14,15].

Ellagic acid (EA) is a naturally occurring polyphenolic compound widely distributed in fruits, nuts, and medicinal plants, particularly pomegranates, berries, walnuts, and oak species. In recent years, EA has attracted considerable attention in veterinary and animal sciences for its potent antioxidant, anti-inflammatory, immunomodulatory, antimicrobial, and cytoprotective properties. These biological activities are primarily attributed to its free radical-scavenging capacity, metal-chelating ability, and regulation of cellular signaling pathways associated with oxidative stress, inflammation, and apoptosis [16,17,18]. Consequently, dietary EA supplementation has been increasingly investigated as a natural functional feed additive to improve animal health, enhance disease resistance, and reduce physiological disturbances induced by environmental and chemical stressors.

In aquaculture, accumulating evidence indicates that dietary EA supplementation has multiple beneficial effects on fish physiology. Previous studies have demonstrated that EA improves growth performance, enhances antioxidant capacity, strengthens innate immune responses, and increases disease resistance in several cultured fish species [19,20,21]. Moreover, EA has been shown to protect fish against oxidative damage induced by environmental contaminants and toxic chemicals, including malathion, chlorpyrifos, ammonia, and developmental oxidative stress, by restoring antioxidant defense systems, reducing lipid peroxidation, and preserving cellular homeostasis [16,22,23,24]. Collectively, these findings suggest that EA is a promising nutritional intervention that enhances physiological resilience to chemically induced stress. Despite growing evidence supporting its protective properties, the potential role of dietary EA in alleviating copper sulfate-induced physiological disturbances in fish has not yet been comprehensively investigated.

Although the beneficial effects of dietary EA have been demonstrated against a variety of environmental pollutants and chemical toxicants, its protective potential against CuSO_4_-induced physiological disturbances in fish has not yet been comprehensively investigated. Moreover, previous studies have primarily focused on pre-exposure supplementation with natural antioxidants, whereas comparative information on the effectiveness of pre-exposure and post-exposure supplementation with EA against CuSO_4_ exposure remains limited. Therefore, the present study was designed to evaluate the effects of pre- and post-exposure dietary EA supplementation on CuSO_4_-induced alterations in hematological characteristics, innate immune responses, and oxidative status in common carp (*Cyprinus carpio*). It was hypothesized that dietary EA would attenuate CuSO_4_-induced physiological disturbances by preserving hematological homeostasis, maintaining innate immune competence, and reinforcing endogenous antioxidant defense systems, thereby enhancing the physiological resilience of fish during CuSO_4_ treatment.

## 2. Materials and Methods

### 2.1. Experimental Fish and Rearing Conditions

Healthy common carp (*Cyprinus carpio*) with an average body weight of approximately 35 g were obtained from a commercial fish farm and transported to the Aquaculture Research and Application Unit, Faculty of Fisheries, Fırat University, Elazig, Türkiye. Upon arrival, fish were acclimated to laboratory conditions for 14 days before the experiment. During acclimation, fish were fed a commercial diet at a daily ration of 5% of body weight, divided into two equal meals. Throughout both the acclimation and experimental periods, fish were maintained in continuously aerated fiberglass tanks supplied with dechlorinated freshwater under controlled environmental conditions. Water quality parameters were monitored regularly and maintained within the optimal range for common carp culture, including a temperature of 24 ± 1 °C, dissolved oxygen of 7.0 ± 0.5 mg/L, pH 7.5 ± 0.2, and total alkalinity of approximately 120 mg CaCO_3_/L. Approximately 30% of the tank water was renewed daily to maintain water quality, and a 12 h light:12 h dark photoperiod was applied throughout the study. Fish were observed daily during the acclimation and experimental periods, and only clinically healthy individuals exhibiting normal swimming and feeding behavior were included in the experiment. All experimental procedures were conducted in accordance with institutional guidelines for the care and use of experimental animals and were approved by the Fırat University Animal Experiments Local Ethics Committee (Approval No: 2026/03–02; Date: 3 February 2026). 

### 2.2. Experimental Diets

EA (≥98% purity, Sigma-Aldrich, St. Louis, MO, USA) was incorporated into a commercial basal diet at 100 mg/kg feed. Due to its limited water solubility, the required amount of EA was first dissolved in a minimal volume of 70% ethanol. The resulting solution was then diluted with distilled water to obtain a sufficient volume for homogeneous application to the feed pellets. The prepared solution was uniformly sprayed onto a commercially available extruded fish diet (Cagatay Oil and Feed Products Inc., Izmir, Türkiye; Ecobio, trout grower feed; proximate composition: 45% crude protein, 20% crude lipid, 11% ash, 3% crude fiber, 8.5% moisture, 12.5% nitrogen-free extract; gross energy: 5124 kcal/kg) while the pellets were continuously mixed to ensure even distribution of EA. After coating, the feed was re-pelleted, spread in a thin layer, and air-dried at room temperature for 48 h to allow complete evaporation of residual ethanol and moisture while preserving the stability of the bioactive compound. The control diet was prepared using the same procedure, except that the EA solution was replaced with an equivalent volume of the ethanol–distilled water vehicle to ensure identical processing conditions among experimental diets. The amount of ethanol used during diet preparation was minimal, and the diets were air-dried before use to allow ethanol evaporation; therefore, no residual solvent effects on the experimental outcomes were expected. All diets were stored in airtight, light-protected containers at 4 °C until use. The dietary inclusion level of EA was selected based on previous studies demonstrating its beneficial effects on antioxidant status, immune responses, and physiological health in fish [19,22,23].

### 2.3. Experimental Design

After the acclimation period, 150 clinically healthy common carp (*Cyprinus carpio*) showing no external signs of disease, injury, or abnormal behavior were individually netted and randomly allocated to five experimental groups, each consisting of three replicate tanks containing 10 fish per tank (30 fish per treatment), to ensure an even distribution of fish among treatment groups and minimize selection bias. The groups were as follows: (1) Control: fish fed the basal diet for 21 consecutive days without copper sulfate exposure; (2) EA: fish fed the EA-supplemented diet (100 mg/kg) for the first 14 consecutive days, then the basal diet for the remaining 7 days without copper sulfate exposure; (3) CuSO_4_: fish fed the basal diet for 21 consecutive days and exposed to CuSO_4_ (1 mg/L, 30 min/day) during the last 7 consecutive days; (4) EA pre-exposure + CuSO_4_: fish fed the EA-supplemented diet (100 mg/kg) for the first 14 consecutive days, then exposed to CuSO_4_ (1 mg/L, 30 min/day) for 7 consecutive days to evaluate the protective effect of pre-exposure EA supplementation; and (5) CuSO_4_ + EA post-exposure: fish exposed to CuSO_4_ (1 mg/L, 30 min/day) for 7 consecutive days, then fed the EA-supplemented diet (100 mg/kg) for 14 consecutive days to evaluate the protective effect of post-exposure EA supplementation. To ensure temporal comparability among the experimental groups, all fish were sampled at the end of the 21-day experimental period. Throughout the experiment, fish were fed their respective diets to apparent satiation twice daily. Feeding activity, survival, and general health were monitored daily, and all fish were carefully observed for abnormal clinical signs. At the end of the experiment, blood and tissue samples were collected for analyses of hematological, innate immune, and oxidative status.

### 2.4. Copper Sulfate Exposure Protocol

Fish were exposed to a nominal concentration of 1 mg/L copper sulfate pentahydrate (CuSO_4_·5H_2_O). Copper sulfate pentahydrate (CuSO_4_·5H_2_O; analytical grade, Merck, Darmstadt, Germany) was freshly dissolved in dechlorinated freshwater immediately before each exposure to achieve a final concentration of 1 mg/L. Fish assigned to the CuSO_4_, EA pre-exposure + CuSO_4_, and CuSO_4_ + EA post-exposure groups were transferred daily to separate exposure tanks containing the prepared CuSO_4_ solution and were maintained under continuous aeration for 30 min. This exposure protocol was repeated once daily for seven consecutive days. Fish were not fed during the exposure period to prevent potential interactions between feed particles and dissolved copper ions and to ensure uniform exposure conditions. Immediately after each exposure, fish were returned to their respective rearing tanks containing clean, well-aerated freshwater, where feeding was resumed according to the experimental protocol. Fresh CuSO_4_ solutions were prepared immediately before each treatment to maintain a constant exposure concentration throughout the study. The exposure concentration was selected based on previous reports describing the therapeutic application and physiological effects of CuSO_4_ in fish, whereas the exposure duration and frequency were established according to the experimental design of the present study [4,5].

### 2.5. Blood and Tissue Sampling

At the end of the experimental period, fish were anesthetized with a 25 mg/L benzocaine solution to minimize handling stress before sampling. Blood samples were collected individually from the caudal vasculature using sterile disposable syringes and immediately divided into three aliquots. Following blood collection, fish were euthanized by decapitation, after which liver, kidney, gill, and spleen tissues were immediately dissected for biochemical analyses. One aliquot was transferred to EDTA-coated tubes for hematological analyses, the second aliquot was placed into heparinized tubes for the determination of nitroblue tetrazolium (NBT) activity, phagocytic activity (PA), and phagocytic index (PI), and the remaining blood was transferred to plain tubes for serum preparation. Serum was obtained by allowing the blood to clot at 4 °C, followed by centrifugation at 3500× *g* for 10 min, and then stored at −80 °C until determination of innate immune parameters. Following blood collection, fish were humanely euthanized, and liver, kidney, gill, and spleen tissues were carefully excised. Tissue samples were rinsed with ice-cold physiological saline (0.9% NaCl) to remove residual blood, gently blotted dry, rapidly frozen in liquid nitrogen, and stored at −80 °C until determination of oxidative stress biomarkers and antioxidant defense parameters. To minimize post-mortem biochemical alterations, all sampling procedures were completed under identical conditions as quickly as possible.

### 2.6. Hematological Analyses

Hematological analyses were performed immediately after blood collection using EDTA-anticoagulated whole blood samples. Red blood cell (RBC) counts were determined with a Neubauer hemocytometer (Isolab, Wertheim, Germany) after dilution with Natt and Herrick’s solution [25]. Hemoglobin (Hb) concentration was measured by the cyanmethemoglobin method using Drabkin’s reagent, and absorbance was recorded at 540 nm [26]. Hematocrit (Ht) was determined by the microhematocrit method after centrifugation of capillary tubes at 12,000× *g* for 5 min [26]. Mean corpuscular volume (MCV), mean corpuscular hemoglobin (MCH), and mean corpuscular hemoglobin concentration (MCHC) were then calculated using standard hematological equations [27].

The erythrocyte indices were calculated as follows:

MCV (µm^3^) = [Ht (%) × 10]/RBC (×10^6^ cells/mm^3^)

MCH (pg) = [Hb (g/dL) × 10]/RBC (×10^6^ cells/mm^3^)

MCHC (%) = [Hb (g/dL) × 100]/Ht (%)

### 2.7. Innate Immune Analyses

Innate immune responses were evaluated by measuring cellular and humoral immune parameters in whole-blood and serum samples. Total white blood cell (WBC) counts were determined simultaneously with erythrocyte counts using Natt and Herrick’s diluting solution and a Neubauer hemocytometer [25]. The respiratory burst activity of circulating phagocytes was assessed using the nitroblue tetrazolium (NBT) reduction assay, following Siwicki et al. [28] with modifications described by İspir and Yonar [29]. The reduction in NBT to formazan was quantified spectrophotometrically at 620 nm and served as an indicator of oxidative burst activity.

Phagocytic activity (PA) and phagocytic index (PI) were determined as described by Siwicki and Anderson [30]. Briefly, heparinized blood samples were incubated with an *Aeromonas hydrophila* suspension (1 × 10^7^ CFU/mL), and Giemsa-stained blood smears were examined under a light microscope. Phagocytic activity was expressed as the percentage of phagocytic cells containing engulfed bacteria, whereas the phagocytic index was defined as the mean number of bacteria ingested per phagocytic cell.

Humoral innate immune responses were evaluated by measuring serum lysozyme (LYZ), myeloperoxidase (MPO), and bactericidal activity (BA). Lysozyme activity was measured using *Micrococcus lysodeikticus* as the substrate, following Ellis [31]. Serum MPO activity was determined according to Quade and Roth [32], with modifications described by Sahoo et al. [33], based on the oxidation of 3,3′,5,5′-tetramethylbenzidine (TMB) by hydrogen peroxide, followed by spectrophotometric measurement at 450 nm. Serum BA was determined according to Kajita et al. [34] by measuring its ability to inhibit the growth of *Aeromonas hydrophila*. Serum TP and IgM levels were determined according to the method described by Siwicki et al. [28].

### 2.8. Oxidative Stress and Antioxidant Analyses

To assess oxidative tissue status, liver, kidney, spleen, and gill samples were processed immediately after thawing on ice. Approximately 0.20–0.25 g of each tissue was homogenized in nine volumes of ice-cold 1.15% potassium chloride (KCl) solution (1:10, *w*/*v*) with a glass homogenizer. The homogenates were centrifuged at 3200 rpm for 10 min at 4 °C, and the supernatants were collected for subsequent biochemical analyses.

Lipid peroxidation was assessed by measuring malondialdehyde (MDA) concentrations via the thiobarbituric acid (TBA) reaction described by Placer et al. [35]. Reduced glutathione (GSH) levels were determined using the method of Ellman [36]. The activities of the antioxidant enzymes superoxide dismutase (SOD), catalase (CAT), glutathione peroxidase (GPx), and glutathione S-transferase (GST) were measured spectrophotometrically using the methods described by Sun et al. [37], Aebi [38], Beutler [39], and Habig et al. [40], respectively. Total protein concentrations in tissue supernatants were determined using the method of Lowry et al. [41], and all biochemical parameters were expressed relative to tissue protein content where appropriate.

### 2.9. Statistical Analysis

All data are presented as the mean ± standard error (SE). Fish were distributed among three tanks per treatment group; however, all hematological, immunological, and oxidative stress biomarkers were determined individually from each sampled fish. Therefore, the reported sample size (*n* = 30) represents individual biological observations obtained from fish maintained under the same treatment conditions. Before statistical analysis, normality of the data distribution was assessed using the Shapiro–Wilk test, and homogeneity of variances was evaluated using Levene’s test. Differences among the five experimental groups were analyzed using one-way analysis of variance (One-Way ANOVA). When significant differences were detected, treatment means were compared using Tukey’s honestly significant difference (HSD) post hoc test. Parametric analyses were performed only after confirming normality and homogeneity of variance. All statistical analyses were conducted using IBM SPSS Statistics for Windows, Version 21.0 (IBM Corp., Armonk, NY, USA). Differences were considered statistically significant at *p* < 0.05.

## 3. Results

### 3.1. Hematological Parameters

Dietary EA supplementation markedly alleviated the hematological disturbances caused by copper sulfate exposure (Table 1). CuSO_4_ exposure significantly reduced RBC count, Hb concentration, Ht, and MCV compared with the control and EA groups (*p* < 0.05). In contrast, MCHC increased significantly after CuSO_4_ exposure, whereas MCH remained unchanged across the experimental groups (*p* > 0.05).

Fish receiving pre-exposure EA supplementation before CuSO_4_ exposure exhibited substantial protection against hematological alterations. RBC, Hb, Ht, and MCV values in the pre-exposure supplementation group were significantly higher than those of the CuSO_4_-treated fish and approached the values observed in the control group (*p* < 0.05). Post-exposure EA supplementation also improved RBC, Hb, and Ht compared with the CuSO_4_ group, whereas MCV did not differ significantly between the two groups. 

Administration of EA alone did not adversely affect any hematological parameter. On the contrary, RBC, Hb, Ht, and MCV values remained comparable to those of the control group, and slight improvements were observed in several erythrocytic indices. Overall, both pre-exposure and post-exposure dietary EA supplementation were associated with improvements in hematological parameters compared with the CuSO_4_ group under the experimental conditions of the present study.

### 3.2. Immunological Parameters

CuSO_4_ exposure significantly affected both cellular and humoral immune responses in common carp (Table 2). Fish exposed to CuSO_4_ exhibited significantly higher WBC, NBT, and MPO values than those of the control and EA groups (*p* < 0.05). In contrast, PA, PI, TP, IgM, BA, and LYZ were significantly reduced following CuSO_4_ exposure (*p* < 0.05).

Pre-exposure EA supplementation markedly alleviated the immunological alterations induced by CuSO_4_. Fish receiving EA before CuSO_4_ exposure showed significantly lower WBC, NBT, and MPO values than those of the CuSO_4_ group, whereas PA, PI, TP, IgM, BA, and LYZ were significantly improved (*p* < 0.05). For most immune parameters, pre-exposure EA supplementation restored values close to those observed in the control group.

Post-exposure EA supplementation was also associated with improvements in immune parameters compared with the CuSO_4_ group. Compared with the post-exposure supplementation group, the pre-exposure supplementation group exhibited lower WBC, NBT, and MPO values and higher PA, PI, TP, IgM, BA, and LYZ values (*p* < 0.05).

Dietary EA administered alone enhanced several immune parameters without inducing adverse effects. Compared with the control group, the EA group exhibited significantly higher PA, PI, TP, IgM, BA, and LYZ values, whereas WBC, NBT, and MPO remained comparable to those of untreated fish (*p* < 0.05). Overall, both pre-exposure and post-exposure dietary EA supplementation were associated with improvements in immunological parameters compared with the CuSO_4_ group under the experimental conditions of the present study.

### 3.3. Oxidative Stress Biomarkers and Antioxidant Responses

CuSO_4_ exposure markedly increased MDA levels across all examined tissues, including the liver, kidney, spleen, and gill (*p* < 0.05; Table 3, Table 4, Table 5 and Table 6). The CuSO_4_ group consistently exhibited the highest MDA concentrations, whereas dietary EA supplementation significantly reduced lipid peroxidation in all tissues. Compared with the post-exposure supplementation group, the pre-exposure supplementation group exhibited lower MDA levels in the examined tissues (*p* < 0.05). In fish receiving dietary EA alone, MDA concentrations remained low and were comparable to those observed in the control group in several tissues. 

A significant reduction in SOD activity was observed in all analyzed tissues following CuSO_4_ exposure (*p* < 0.05). Dietary EA supplementation effectively restored SOD activity in all examined tissues. Compared with the post-exposure supplementation group, the pre-exposure supplementation group exhibited higher SOD activity in most of the examined tissues, although this pattern was not observed in every tissue. Fish receiving dietary EA alone exhibited high SOD activity, whereas the CuSO_4_ group consistently showed the lowest SOD activity across all tissues examined.

CAT activity followed a pattern similar to SOD. CuSO_4_ exposure significantly suppressed CAT activity in the liver, kidney, spleen, and gill compared with the control and EA groups (*p* < 0.05). Both pre-exposure and post-exposure EA supplementation significantly improved CAT activity. Compared with the post-exposure supplementation group, the pre-exposure supplementation group exhibited higher CAT activity, with values closer to those of the Control group in most examined tissues.

GPx activity was significantly reduced by CuSO_4_ exposure across all examined tissues (*p* < 0.05). Dietary EA supplementation markedly alleviated these reductions. Compared with the post-exposure supplementation group, the pre-exposure supplementation group exhibited higher GPx activity in the examined tissues. Across all tissues, the EA group exhibited the highest GPx activity, whereas the CuSO_4_ group showed the lowest.

GST activity was also significantly affected by the experimental treatments (*p* < 0.05). CuSO_4_ exposure markedly reduced GST activity across all tissues compared with the control and EA groups. Dietary EA supplementation preserved GST activity. Compared with the post-exposure supplementation group, the pre-exposure supplementation group exhibited higher GST activity in the examined tissues. No significant differences were observed between the control and EA groups in the liver and kidney, whereas the EA group showed slightly higher GST activity than the control group in the spleen and gill.

GSH concentrations were significantly depleted in all analyzed tissues after CuSO_4_ exposure compared with the control and EA groups (*p* < 0.05). Dietary EA supplementation effectively restored GSH levels. Compared with the post-exposure supplementation group, the pre-exposure supplementation group exhibited higher GSH levels in the examined tissues. Fish fed the EA-supplemented diet alone exhibited the highest GSH concentrations, whereas the CuSO_4_ group consistently showed the lowest levels across all tissues.

## 4. Discussion

CuSO_4_ remains one of the most widely used chemotherapeutic agents in aquaculture because of its broad-spectrum antiparasitic, antifungal, and antimicrobial activities. However, its application is limited by its potential to induce oxidative stress and disrupt normal physiological functions in fish, particularly after repeated or prolonged exposure. Therefore, identifying safe and effective nutritional strategies to minimize these adverse effects has become an important research priority. In the present study, dietary EA supplementation markedly alleviated CuSO_4_-induced hematological, immunological, and oxidative disturbances in common carp. Moreover, both pre-exposure and post-exposure EA supplementation were associated with marked improvements in hematological, immunological, and oxidative stress responses under the experimental conditions of the present study.

Hematological parameters are widely recognized as sensitive and reliable indicators of fish health because they rapidly reflect physiological disturbances induced by environmental stressors, toxicants, and disease conditions. Consequently, hematological biomarkers are extensively used to assess the physiological consequences of copper exposure and the effectiveness of protective interventions in fish [8,27]. In the present study, CuSO_4_ exposure significantly disrupted hematological homeostasis in common carp, as evidenced by reductions in RBC, Hb, Ht, and MCV together with an increase in MCHC. These findings are consistent with previous reports describing CuSO_4_-induced hematological disturbances in common carp (*Cyprinus carpio*) [42,43], Nile tilapia (*Oreochromis niloticus*) [44,45,46], rohu (*Labeo rohita*) [8], rainbow trout (*Oncorhynchus mykiss*) [47], and other freshwater fish species [48,49]. Likewise, Tavares-Dias [5] identified hematological variables as among the most sensitive biomarkers of copper sulfate toxicity in fish. Excess copper has been reported to promote the generation of reactive oxygen species (ROS) via redox cycling, which may lead to oxidative damage to erythrocyte membranes, hemoglobin oxidation, membrane fragility, and hemolysis. These alterations are thought to impair oxygen transport and may contribute to the reductions in RBC, Hb, and Ht, while changes in erythrocyte morphology may be reflected in erythrocyte indices.

The marked improvement in hematological parameters following dietary EA supplementation is consistent with the well-documented hematoprotective properties of this natural polyphenol. Fish receiving EA supplementation exhibited significant restoration of RBC, Hb, Ht, and MCV compared with the CuSO_4_ group following both pre-exposure and post-exposure EA supplementation. Differences between the two supplementation strategies were observed; however, these should be interpreted in light of the experimental design and the absence of a time-matched CuSO_4_ recovery control group. Similar beneficial effects have been reported in rainbow trout, where dietary EA improved hematological parameters under both normal rearing conditions and stress challenges [19,50]. Likewise, dietary EA supplementation enhanced growth and health performance in Nile tilapia [21] and improved growth performance and antioxidant status in common carp [20], further supporting its role in maintaining physiological homeostasis. Comparable hematoprotective effects have also been demonstrated in mammalian models exposed to sodium arsenite and lead, where EA alleviated toxicant-induced hematological impairments [51,52]. Furthermore, Witeska et al. [27] emphasized that preservation of erythrocyte integrity and hematopoietic function is a reliable indicator of improved physiological status in fish exposed to environmental stressors. The hematoprotective effects of EA may be attributable to its antioxidant activity, preservation of erythrocyte membrane integrity, attenuation of oxidative injury, and maintenance of normal hematopoietic function, as suggested by previous studies. These proposed mechanisms may contribute to improved erythrocyte survival, oxygen-carrying capacity, and restoration of hematological homeostasis during CuSO_4_-induced stress; however, they were not directly investigated in the present study.

The innate immune system constitutes the primary defense mechanism against invading pathogens and environmental stressors in fish and plays a pivotal role in maintaining health and disease resistance. Cellular and humoral immune parameters, including phagocytic activity, respiratory burst, lysozyme activity, bactericidal activity, and immunoglobulin levels, are widely used as reliable biomarkers of immunocompetence and for evaluating immunotoxicity and the efficacy of dietary immunomodulators in aquaculture [53,54,55]. In the present study, CuSO_4_ exposure markedly disrupted both cellular and humoral immune responses in common carp, as indicated by significant increases in WBC, NBT, and MPO together with reductions in PA, PI, TP, IgM, BA, and LYZ. This response pattern suggests enhanced leukocyte activation and oxidative burst accompanied by impaired phagocytic and antimicrobial functions. Increased NBT and MPO activities indicate intensified respiratory burst, whereas the concurrent reductions in PA and PI demonstrate diminished phagocytic efficiency. Likewise, decreased TP, IgM, BA, and LYZ indicate suppression of humoral and nonspecific antimicrobial defenses. Similar immunological disturbances following copper exposure have been reported in fish, including intestinal inflammation, oxidative injury, and impaired tissue integrity [9,10,56,57], altered inflammatory and antioxidant gene expression in common carp [14], and changes in gill-associated molecular responses and host microbiota in yellow catfish [11]. Comparable inflammatory and oxidative alterations have also been documented in zebrafish and other aquatic models exposed to copper-related stress [58,59]. Tavares-Dias [5] likewise emphasized that the immunological consequences of CuSO_4_ depend on exposure conditions, fish species, and environmental factors but may ultimately reduce resistance to secondary stressors. Collectively, these findings indicate that the simultaneous elevation of WBC, NBT, and MPO together with suppression of PA, PI, TP, IgM, BA, and LYZ reflects a dysregulated stress-associated immune response rather than beneficial immunostimulation. At the molecular level, previous studies have suggested that excess copper may promote ROS accumulation and activation of redox-sensitive inflammatory pathways, particularly NF-κB, while disrupting Nrf2-dependent antioxidant regulation, thereby impairing leukocyte function and antimicrobial immunity [60]. However, these molecular mechanisms were not directly investigated in the present study and are presented here as plausible explanations based on previous literature.

Dietary EA supplementation alone enhanced the immune status of common carp, as reflected by increases in PA, PI, TP, IgM, BA, and LYZ, whereas WBC, NBT, and MPO remained close to Control levels. This pattern suggests that EA improved both cellular and humoral immune competence without inducing excessive leukocyte activation or oxidative burst. Similar immunostimulatory effects have been reported in rainbow trout [19,50], Nile tilapia [21], and sterlet [55], where dietary EA or ellagitannin-based interventions improved nonspecific immune responses and general health. Evidence from mammalian and cellular models further indicates that EA modulates macrophage activity, cytokine production, innate immune mediators, and mucosal defense rather than simply enhancing inflammatory responses [53,61,62,63]. These findings support the role of EA as an immunomodulatory compound that strengthens protective immune functions while maintaining inflammatory balance. In the present study, EA also effectively counteracted CuSO_4_-induced immune dysfunction. Compared with the CuSO_4_ group, EA supplementation reduced the excessive elevations in WBC, NBT, and MPO while restoring PA, PI, TP, IgM, BA, and LYZ. Both pre-exposure and post-exposure EA supplementation markedly improved immune responses compared with the CuSO_4_ group. Differences between the two supplementation strategies were observed under the experimental conditions of the present study. These findings suggest that dietary EA supplementation may contribute to maintaining antioxidant and immune competence during experimental CuSO_4_ exposure. The protective effects of EA may involve coordinated regulation of oxidative and inflammatory pathways, as suggested by previous studies. Previous studies have suggested that EA may suppress NF-κB activation, modulate cytokine balance and macrophage function, enhance antioxidant-dependent anti-inflammatory signaling [64], regulate innate immune mediators [53,65,66], and preserve intestinal integrity, microbiota balance, and mucosal immunity [17,67,68]. Collectively, these proposed mechanisms may contribute to protection against CuSO_4_-induced immunotoxicity by limiting ROS-driven inflammatory activation, preventing excessive respiratory burst, preserving phagocyte function, and maintaining humoral and antimicrobial defenses. However, these mechanisms were not directly investigated in the present study and are presented as hypotheses based on previous literature.

Oxidative stress is one of the principal mechanisms underlying toxicant-induced cellular injury in fish. Excessive production of reactive oxygen species (ROS) has been reported to disrupt cellular redox homeostasis, which may lead to lipid peroxidation, protein oxidation, DNA damage, and functional impairment of biological macromolecules [69,70,71]. Fish counteract these effects through an integrated antioxidant defense system comprising enzymatic antioxidants (SOD, CAT, GPx, and GST) and the non-enzymatic antioxidant GSH. Accordingly, oxidative stress biomarkers and antioxidant enzymes are widely accepted as sensitive indicators of toxicological responses and antioxidant efficacy in aquatic organisms [72,73,74]. In the present study, CuSO_4_ exposure markedly disrupted redox homeostasis in common carp, as evidenced by increased MDA and decreased SOD, CAT, GPx, GST, and GSH levels in the liver, kidney, spleen, and gill. Elevated MDA is indicative of enhanced lipid peroxidation, whereas the simultaneous depletion of enzymatic and non-enzymatic antioxidant defenses suggests that CuSO_4_-induced oxidative stress may have exceeded the capacity of endogenous protective systems. These findings are consistent with previous studies demonstrating that copper exposure induces tissue-specific oxidative damage, antioxidant depletion, and histopathological injury in fish [75,76,77]. Similar increases in oxidative stress markers and reductions in antioxidant capacity have also been reported in Nile tilapia, rohu, common carp, yellowtail kingfish, and yellow catfish exposed to copper or CuSO_4_ [8,11,78,79,80].

The observed decreases in SOD and CAT suggest impairment of the first-line antioxidant defense system responsible for detoxifying superoxide radicals and removing hydrogen peroxide. Likewise, reductions in GPx, GST, and GSH suggest a weakened glutathione-dependent defense network, limiting the detoxification of lipid hydroperoxides and reactive electrophilic metabolites. Similar disturbances in antioxidant enzyme activity and non-enzymatic antioxidant reserves have been reported in the brain, muscle, gill, liver, and blood cells of fish following copper exposure [77,81,82,83]. The pronounced MDA accumulation observed in the gill is biologically plausible because this tissue represents the primary interface between dissolved copper and the fish and is therefore directly exposed before systemic distribution. Previous studies have suggested that, at the molecular level, excess copper may promote ROS generation via redox cycling, leading to the oxidation of membrane lipids, proteins, and nucleic acids. Copper exposure has also been reported to disrupt Nrf2/ARE-mediated antioxidant signaling, thereby reducing the transcriptional activation of cytoprotective enzymes and weakening cellular resistance to oxidative injury [81,82]. In parallel, oxidative stress may amplify inflammatory and apoptotic pathways, aggravating tissue damage and delaying physiological recovery [10,14]. Collectively, the simultaneous increase in MDA and depletion of SOD, CAT, GPx, GST, and GSH suggest that CuSO_4_ induced marked redox imbalance in common carp. Based on previous studies, this response may involve excessive ROS generation, suppression of endogenous antioxidant defenses, and disruption of Nrf2-dependent cytoprotective signaling. However, these molecular mechanisms were not directly investigated in the present study and are presented as plausible explanations based on previous literature.

Dietary EA supplementation alone markedly improved tissue redox status, as evidenced by lower MDA levels and higher SOD, CAT, GPx, GST, and GSH levels in the liver, kidney, spleen, and gill. This pattern indicates that EA reduced basal lipid peroxidation while strengthening both enzymatic and non-enzymatic antioxidant defenses. Similar improvements have been reported in common carp, rainbow trout, Nile tilapia, and yellow catfish, where dietary EA enhanced antioxidant capacity, reduced oxidative damage, and improved physiological resilience under normal or stressful conditions [19,20,21,24]. Comparable protective effects were also observed in common carp exposed to malathion or chlorpyrifos, with EA reducing lipid peroxidation and restoring antioxidant parameters [22,23]. In zebrafish embryos, EA likewise attenuated oxidative injury and preserved developmental integrity [16]. These findings support the role of EA as a broad-spectrum antioxidant that preserves redox homeostasis under both physiological and toxicological conditions. In the present study, EA also effectively counteracted the oxidative injury induced by CuSO_4_. Compared with the CuSO_4_ group, EA supplementation reduced MDA accumulation and restored SOD, CAT, GPx, GST, and GSH in all examined tissues. Both pre-exposure and post-exposure EA supplementation were associated with significant improvements in oxidative stress biomarkers compared with the CuSO_4_ group. Differences between the two supplementation strategies were observed; however, these should be interpreted in light of the experimental design and the absence of a time-matched CuSO_4_ recovery control group. However, because the pre-exposure and post-exposure protocols were designed to evaluate two different intervention strategies rather than a direct time-matched comparison, the observed differences between these two approaches should be interpreted with caution. The protective effects of EA may involve multiple complementary mechanisms, including direct scavenging of reactive oxygen and nitrogen species, chelation of redox-active metal ions, interruption of lipid peroxidation chain reactions, and stabilization of cellular membranes [72,73,84]. In addition, EA and its urolithin metabolites have been reported to modulate endogenous antioxidant signaling and improve cellular resistance to oxidative stress [85]. EA has also been reported to activate Nrf2-dependent pathways, thereby promoting the expression of antioxidant and cytoprotective enzymes while reducing ROS accumulation [86]. Similar beneficial effects on antioxidant activity, intestinal integrity, inflammation, and lipid metabolism have been reported in piglets, broilers, and mice [87,88,89,90]. Collectively, these findings suggest that the protective effects of EA against CuSO_4_-induced oxidative toxicity may involve multiple complementary mechanisms, including direct radical scavenging, metal chelation, inhibition of lipid peroxidation, preservation of the glutathione-dependent defense system, and modulation of Nrf2-mediated antioxidant responses, as proposed in previous studies. However, these mechanisms were not directly investigated in the present study and therefore should be regarded as plausible hypotheses rather than experimentally confirmed pathways.

## 5. Conclusions

The present study demonstrated that repeated CuSO_4_ exposure induced pronounced hematological, immunological, and oxidative alterations in common carp, indicating that repeated exposure may adversely affect physiological status under the experimental conditions used. Dietary EA supplementation effectively alleviated these adverse effects by restoring hematological homeostasis, improving innate immune responses, reducing lipid peroxidation, and reinforcing enzymatic and non-enzymatic antioxidant defenses. Both pre-exposure and post-exposure EA supplementation were associated with improvements in hematological, immunological, and oxidative stress biomarkers following experimental CuSO_4_ exposure. Under the experimental conditions of the present study, pre-exposure supplementation generally produced more favorable responses than post-exposure supplementation. These findings suggest that dietary EA may be a promising natural functional feed additive for supporting hematological, immunological, and antioxidant status during experimental CuSO_4_ exposure in common carp. Further studies incorporating pathogen challenge models are required to determine whether EA influences the therapeutic efficacy of CuSO_4_ under practical aquaculture conditions. Although the present study demonstrated significant protective effects of dietary EA at 100 mg/kg, future dose–response studies evaluating multiple dietary inclusion levels are warranted to determine the optimal supplementation level for practical aquaculture applications. Future studies investigating the molecular mechanisms underlying the protective actions of EA, particularly its potential interactions with redox-sensitive signaling pathways and immune regulation, will help clarify its mode of action and further support its application in sustainable aquaculture and aquatic veterinary medicine.

## 6. Study Limitations

One limitation of the present study is the absence of a CuSO_4_-exposed recovery control group maintained on the basal diet during the post-exposure period. Consequently, although post-exposure EA supplementation was associated with marked improvements in hematological, immunological, oxidative stress, and histopathological parameters, the contribution of spontaneous recovery following the cessation of CuSO_4_ exposure cannot be completely excluded. The primary objective of this study was to compare pre-exposure and post-exposure EA supplementation strategies rather than to investigate the natural recovery process after copper withdrawal. Therefore, future studies incorporating a time-matched CuSO_4_ recovery group without EA supplementation would help distinguish the effects of EA from spontaneous recovery.

A further limitation relates to the statistical treatment of individually measured biomarkers obtained from fish maintained in replicate tanks. Although hematological, immunological, oxidative stress, and histopathological parameters were measured individually from each fish, potential tank-related clustering was not explicitly incorporated into the statistical analysis. The present study was designed to evaluate individual biological responses to the experimental treatments; however, future studies including larger numbers of independent tank replicates and retaining tank identity for each individual observation would facilitate the application of hierarchical or mixed-effects statistical models to further evaluate potential tank-level variability and strengthen statistical inference.

## Figures and Tables

**Table 1:** Effects of pre-exposure and post-exposure dietary EA supplementation on hematological parameters of common carp (*Cyprinus carpio*) exposed to copper sulfate (CuSO_4_).

	Groups				
Parameters	Control	EA	CuSO_4_	EA + CuSO_4_	CuSO_4_ + EA
RBC	1.49 ± 0.18 ^ab^	1.52 ± 0.21 ^a^	1.16 ± 0.13 ^d^	1.45 ± 0.15 ^b^	1.31 ± 0.22 ^c^
Hb	7.11 ± 0.23 ^a^	7.19 ± 0.17 ^a^	5.54 ± 0.26 ^c^	6.98 ± 0.21 ^a^	6.27 ± 0.13 ^b^
Ht	32.68 ± 2.42 ^a^	34.03 ± 3.10 ^a^	21.87 ± 2.33 ^c^	31.10 ± 2.56 ^a^	25.03 ± 2.69 ^b^
MCV	218.67 ± 11.49 ^a^	222.96 ± 9.78 ^a^	189.03 ± 8.46 ^b^	215.48 ± 6.66 ^a^	192.35 ± 7.30 ^b^
MCH	47.61 ± 3.72 ^a^	47.58 ± 2.61 ^a^	47.97 ± 3.87 ^a^	48.72 ± 5.04 ^a^	47.62 ± 3.47 ^a^
MCHC	21.19 ± 2.14 ^a^	21.08 ± 2.09 ^a^	25.67 ± 3.16 ^b^	22.35 ± 3.38 ^a^	25.75 ± 2.81 ^b^

Data are presented as mean ± SE (*n* = 30). Different superscript letters within the same row indicate significant differences among treatment groups according to a one-way analysis of variance (ANOVA) followed by Tukey’s honestly significant difference (HSD) post hoc test (*p* < 0.05). Control: fish fed the basal diet for 21 consecutive days; EA: fish fed a diet supplemented with 100 mg ellagic acid (EA)/kg feed for the first 14 consecutive days, followed by the basal diet for the remaining 7 days; CuSO_4_: fish fed the basal diet for 21 consecutive days and exposed to copper sulfate (1 mg/L, 30 min/day) during the final 7 consecutive days; EA + CuSO_4_: fish fed the EA-supplemented diet (100 mg/kg feed) for the first 14 consecutive days, followed by CuSO_4_ exposure (1 mg/L, 30 min/day) for 7 consecutive days (pre-exposure supplementation); CuSO_4_ + EA: fish exposed to CuSO_4_ (1 mg/L, 30 min/day) for the first 7 consecutive days, followed by dietary EA supplementation (100 mg/kg feed) for the remaining 14 consecutive days (post-exposure supplementation). RBC: Red blood cell (×10^6^); Hb: Hemoglobin (g/dL); Ht: Hematocrit (%); MCV: Mean corpuscular volume (µm^3^); MCH: Mean corpuscular hemoglobin (pg); MCHC: Mean corpuscular hemoglobin concentration (%).

**Table 2:** Effects of pre-exposure and post-exposure dietary EA supplementation on immunological parameters of common carp (*Cyprinus carpio*) exposed to copper sulfate (CuSO_4_).

	Groups				
Parameters	Control	EA	CuSO_4_	EA + CuSO_4_	CuSO_4_ + EA
WBC	35.69 ± 2.87 ^c^	36.10 ± 3.11 ^c^	46.88 ± 3.42 ^a^	37.52 ± 2.44 ^c^	41.42 ± 2.18 ^b^
NBT	1.27 ± 0.23 ^c^	1.29 ± 0.19 ^c^	1.71 ± 0.28 ^a^	1.33 ± 0.21 ^c^	1.49 ± 0.23 ^b^
PA	33.75 ± 2.55 ^b^	38.50 ± 2.75 ^a^	26.75 ± 2.25 ^d^	34.10 ± 1.95 ^b^	30.04 ± 2.17 ^c^
PI	3.30 ± 0.21 ^c^	4.10 ± 0.35 ^a^	2.57 ± 0.14 ^e^	3.75 ± 0.33 ^b^	2.98 ± 0.20 ^d^
TP	31.56 ± 2.29 ^b^	36.49 ± 3.08 ^a^	22.03 ± 2.59 ^d^	29.96 ± 1.62 ^bc^	27.03 ± 2.06 ^c^
IgM	15.07 ± 2.63 ^b^	22.76 ± 1.99 ^a^	9.98 ± 2.06 ^c^	14.18 ± 1.43 ^b^	13.86 ± 1.99 ^b^
BA	38.75 ± 3.08 ^b^	45.45 ± 4.32 ^a^	30.26 ± 2.94 ^c^	36.91 ± 1.85 ^b^	35.24 ± 3.22 ^b^
LYZ	132.10 ± 9.20 ^b^	146.22 ± 7.63 ^a^	92.75 ± 5.08 ^d^	128.87 ± 8.53 ^bc^	119.30 ± 6.65 ^c^
MPO	0.75 ± 0.16 ^c^	0.77 ± 0.20 ^c^	1.11 ± 0.17 ^a^	0.78 ± 0.24 ^c^	0.95 ± 0.19 ^b^

Data are presented as mean ± SE (*n* = 30). Different superscript letters within the same row indicate significant differences among treatment groups according to a one-way analysis of variance (ANOVA) followed by Tukey’s honestly significant difference (HSD) post hoc test (*p* < 0.05). Control: fish fed the basal diet for 21 consecutive days; EA: fish fed a diet supplemented with 100 mg ellagic acid (EA)/kg feed for the first 14 consecutive days, followed by the basal diet for the remaining 7 days; CuSO_4_: fish fed the basal diet for 21 consecutive days and exposed to copper sulfate (1 mg/L, 30 min/day) during the final 7 consecutive days; EA + CuSO_4_: fish fed the EA-supplemented diet (100 mg/kg feed) for the first 14 consecutive days, followed by CuSO_4_ exposure (1 mg/L, 30 min/day) for 7 consecutive days (pre-exposure supplementation); CuSO_4_ + EA: fish exposed to CuSO_4_ (1 mg/L, 30 min/day) for the first 7 consecutive days, followed by dietary EA supplementation (100 mg/kg feed) for the remaining 14 consecutive days (post-exposure supplementation). WBC: White blood cell counts (×10^3^); NBT: Nitroblue tetrazolium assay (mg/mL); PA: Phagocytic activity (%); PI: Phagocytic index; TP: Total protein level (mg/mL); IgM: Total immunoglobulin M level (mg/mL); BA: Serum bactericidal activity (% cfu/control); LYZ: Lysozyme activity (U/mL); MPO: Myeloperoxidase activity (Optic density).

**Table 3:** Effects of pre-exposure and post-exposure dietary EA supplementation on oxidative stress biomarkers and antioxidant status in the liver of common carp (*Cyprinus carpio*) exposed to copper sulfate (CuSO_4_).

	Groups				
Parameters	Control	EA	CuSO_4_	EA + CuSO_4_	CuSO_4_ + EA
MDA	1.33 ± 0.14 ^d^	1.06 ± 0.11 ^e^	3.68 ± 0.43 ^a^	1.86 ± 0.32 ^c^	2.25 ± 0.27 ^b^
SOD	2.92 ± 0.23 ^b^	3.26 ± 0.41 ^a^	1.64 ± 0.32 ^e^	2.55 ± 0.27 ^c^	2.13 ± 0.18 ^d^
CAT	3.23 ± 0.47 ^b^	3.79 ± 0.32 ^a^	1.76 ± 0.25 ^d^	3.11 ± 0.39 ^b^	2.85 ± 0.51 ^c^
GPx	2.61 ± 0.19 ^b^	3.02 ± 0.24 ^a^	1.23 ± 0.14 ^e^	2.24 ± 0.30 ^c^	1.90 ± 0.23 ^d^
GST	104.08 ± 6.24 ^a^	101.59 ± 5.03 ^a^	66.24 ± 8.87 ^c^	93.51 ± 6.41 ^ab^	87.33 ± 4.89 ^b^
GSH	79.53 ± 4.49 ^b^	91.42 ± 3.46 ^a^	51.03 ± 3.30 ^d^	76.85 ± 4.06 ^b^	65.17 ± 3.72 ^c^

Data are presented as mean ± SE (*n* = 30). Different superscript letters within the same row indicate significant differences among treatment groups according to a one-way analysis of variance (ANOVA) followed by Tukey’s honestly significant difference (HSD) post hoc test (*p* < 0.05). Control: fish fed the basal diet for 21 consecutive days; EA: fish fed a diet supplemented with 100 mg ellagic acid (EA)/kg feed for the first 14 consecutive days, followed by the basal diet for the remaining 7 days; CuSO_4_: fish fed the basal diet for 21 consecutive days and exposed to copper sulfate (1 mg/L, 30 min/day) during the final 7 consecutive days; EA + CuSO_4_: fish fed the EA-supplemented diet (100 mg/kg feed) for the first 14 consecutive days, followed by CuSO_4_ exposure (1 mg/L, 30 min/day) for 7 consecutive days (pre-exposure supplementation); CuSO_4_ + EA: fish exposed to CuSO_4_ (1 mg/L, 30 min/day) for the first 7 consecutive days, followed by dietary EA supplementation (100 mg/kg feed) for the remaining 14 consecutive days (post-exposure supplementation). MDA: Malondialdehyde (nmol/mg protein); SOD: Superoxide dismutase (U/mg protein); CAT: Catalase (k/mg protein); GPx: Glutathione peroxidase (U/mg protein); GST: Glutathione S-transferase (U/mg protein); GSH: Reduced glutathione (µmol/mg protein).

**Table 4:** Effects of pre-exposure and post-exposure dietary EA supplementation on oxidative stress biomarkers and antioxidant status in the kidney of common carp (*Cyprinus carpio*) exposed to copper sulfate (CuSO_4_).

	Groups				
Parameters	Control	EA	CuSO_4_	EA + CuSO_4_	CuSO_4_ + EA
MDA	1.78 ± 0.39 ^d^	1.67 ± 0.21 ^d^	4.05 ± 0.49 ^a^	2.07 ± 0.18 ^c^	2.44 ± 0.30 ^b^
SOD	2.57 ± 0.19 ^b^	2.95 ± 0.33 ^a^	1.63 ± 0.27 ^e^	2.22 ± 0.14 ^c^	1.99 ± 0.16 ^d^
CAT	2.96 ± 0.28 ^b^	3.24 ± 0.28 ^a^	1.81 ± 0.30 ^e^	2.63 ± 0.19 ^c^	2.24 ± 0.25 ^d^
GPx	2.35 ± 0.16 ^b^	2.73 ± 0.35 ^a^	1.47 ± 0.19 ^e^	2.02 ± 0.11 ^c^	1.76 ± 0.21 ^d^
GST	91.11 ± 4.72 ^a^	90.78 ± 5.41 ^a^	63.22 ± 6.02 ^c^	88.96 ± 4.17 ^a^	74.35 ± 4.45 ^b^
GSH	65.24 ± 3.67 ^b^	76.43 ± 4.86 ^a^	42.84 ± 3.18 ^e^	58.08 ± 3.22 ^c^	52.43 ± 3.35 ^d^

Data are presented as mean ± SE (*n* = 30). Different superscript letters within the same row indicate significant differences among treatment groups according to a one-way analysis of variance (ANOVA) followed by Tukey’s honestly significant difference (HSD) post hoc test (*p* < 0.05). Control: fish fed the basal diet for 21 consecutive days; EA: fish fed a diet supplemented with 100 mg ellagic acid (EA)/kg feed for the first 14 consecutive days, followed by the basal diet for the remaining 7 days; CuSO_4_: fish fed the basal diet for 21 consecutive days and exposed to copper sulfate (1 mg/L, 30 min/day) during the final 7 consecutive days; EA + CuSO_4_: fish fed the EA-supplemented diet (100 mg/kg feed) for the first 14 consecutive days, followed by CuSO_4_ exposure (1 mg/L, 30 min/day) for 7 consecutive days (pre-exposure supplementation); CuSO_4_ + EA: fish exposed to CuSO_4_ (1 mg/L, 30 min/day) for the first 7 consecutive days, followed by dietary EA supplementation (100 mg/kg feed) for the remaining 14 consecutive days (post-exposure supplementation). MDA: Malondialdehyde (nmol/mg protein); SOD: Superoxide dismutase (U/mg protein); CAT: Catalase (k/mg protein); GPx: Glutathione peroxidase (U/mg protein); GST: Glutathione S-transferase (U/mg protein); GSH: Reduced glutathione (µmol/mg protein).

**Table 5:** Effects of pre-exposure and post-exposure dietary EA supplementation on oxidative stress biomarkers and antioxidant status in the spleen of common carp (*Cyprinus carpio*) exposed to copper sulfate (CuSO_4_).

	Groups				
Parameters	Control	EA	CuSO_4_	EA + CuSO_4_	CuSO_4_ + EA
MDA	1.58 ± 0.29 ^d^	1.51 ± 0.24 ^d^	3.12 ± 0.66 ^a^	1.95 ± 0.52 ^c^	2.28 ± 0.35 ^b^
SOD	2.35 ± 0.18 ^a^	2.44 ± 0.28 ^a^	1.39 ± 0.15 ^d^	2.03 ± 0.24 ^b^	1.76 ± 0.19 ^c^
CAT	2.48 ± 0.24 ^a^	2.57 ± 0.43 ^a^	1.53 ± 0.30 ^d^	2.24 ± 0.16 ^b^	2.00 ± 0.22 ^c^
GPx	2.19 ± 0.16 ^a^	2.31 ± 0.26 ^a^	1.24 ± 0.15 ^d^	1.98 ± 0.14 ^b^	1.72 ± 0.29 ^c^
GST	79.13 ± 3.43 ^a^	82.07 ± 4.51 ^a^	59.28 ± 3.97 ^c^	76.07 ± 3.49 ^a^	68.68 ± 2.76 ^b^
GSH	61.03 ± 3.66 ^b^	69.15 ± 4.20 ^a^	43.71 ± 2.72 ^d^	58.99 ± 3.06 ^b^	52.86 ± 2.10 ^c^

Data are presented as mean ± SE (*n* = 30). Different superscript letters within the same row indicate significant differences among treatment groups according to a one-way analysis of variance (ANOVA) followed by Tukey’s honestly significant difference (HSD) post hoc test (*p* < 0.05). Control: fish fed the basal diet for 21 consecutive days; EA: fish fed a diet supplemented with 100 mg ellagic acid (EA)/kg feed for the first 14 consecutive days, followed by the basal diet for the remaining 7 days; CuSO_4_: fish fed the basal diet for 21 consecutive days and exposed to copper sulfate (1 mg/L, 30 min/day) during the final 7 consecutive days; EA + CuSO_4_: fish fed the EA-supplemented diet (100 mg/kg feed) for the first 14 consecutive days, followed by CuSO_4_ exposure (1 mg/L, 30 min/day) for 7 consecutive days (pre-exposure supplementation); CuSO_4_ + EA: fish exposed to CuSO_4_ (1 mg/L, 30 min/day) for the first 7 consecutive days, followed by dietary EA supplementation (100 mg/kg feed) for the remaining 14 consecutive days (post-exposure supplementation). MDA: Malondialdehyde (nmol/mg protein); SOD: Superoxide dismutase (U/mg protein); CAT: Catalase (k/mg protein); GPx: Glutathione peroxidase (U/mg protein); GST: Glutathione S-transferase (U/mg protein); GSH: Reduced glutathione (µmol/mg protein).

**Table 6:** Effects of pre-exposure and post-exposure dietary EA supplementation on oxidative stress biomarkers and antioxidant status in the gill of common carp (*Cyprinus carpio*) exposed to copper sulfate (CuSO_4_).

	Groups				
Parameters	Control	EA	CuSO_4_	EA + CuSO_4_	CuSO_4_ + EA
MDA	1.27 ± 0.18 ^d^	1.28 ± 0.15 ^d^	5.41 ± 0.63 ^a^	2.59 ± 0.40 ^c^	3.11 ± 0.34 ^b^
SOD	1.42 ± 0.15 ^a^	1.45 ± 0.22 ^a^	0.71 ± 0.11 ^d^	1.05 ± 0.29 ^c^	1.26 ± 0.20 ^b^
CAT	1.68 ± 0.26 ^a^	1.74 ± 0.33 ^a^	0.96 ± 0.14 ^d^	1.45 ± 0.20 ^b^	1.19 ± 0.11 ^c^
GPx	1.32 ± 0.11 ^b^	1.48 ± 0.17 ^a^	0.63 ± 0.09 ^d^	1.28 ± 0.32 ^b^	1.02 ± 0.13 ^c^
GST	70.71 ± 4.98 ^ab^	74.13 ± 3.41 ^a^	48.06 ± 2.12 ^d^	68.33 ± 3.09 ^b^	59.76 ± 2.87 ^c^
GSH	64.27 ± 3.49 ^b^	76.28 ± 4.52 ^a^	41.29 ± 3.10 ^d^	62.46 ± 4.36 ^b^	53.87 ± 2.45 ^c^

Data are presented as mean ± SE (*n* = 30). Different superscript letters within the same row indicate significant differences among treatment groups according to a one-way analysis of variance (ANOVA) followed by Tukey’s honestly significant difference (HSD) post hoc test (*p* < 0.05). Control: fish fed the basal diet for 21 consecutive days; EA: fish fed a diet supplemented with 100 mg ellagic acid (EA)/kg feed for the first 14 consecutive days, followed by the basal diet for the remaining 7 days; CuSO_4_: fish fed the basal diet for 21 consecutive days and exposed to copper sulfate (1 mg/L, 30 min/day) during the final 7 consecutive days; EA + CuSO_4_: fish fed the EA-supplemented diet (100 mg/kg feed) for the first 14 consecutive days, followed by CuSO_4_ exposure (1 mg/L, 30 min/day) for 7 consecutive days (pre-exposure supplementation); CuSO_4_ + EA: fish exposed to CuSO_4_ (1 mg/L, 30 min/day) for the first 7 consecutive days, followed by dietary EA supplementation (100 mg/kg feed) for the remaining 14 consecutive days (post-exposure supplementation). MDA: Malondialdehyde (nmol/mg protein); SOD: Superoxide dismutase (U/mg protein); CAT: Catalase (k/mg protein); GPx: Glutathione peroxidase (U/mg protein); GST: Glutathione S-transferase (U/mg protein); GSH: Reduced glutathione (µmol/mg protein).

## Data Availability

The original contributions presented in this study are included in the article. Further inquiries can be directed to the corresponding author(s).
